# HandFI: Multilevel Interacting Hand Reconstruction Based on Multilevel Feature Fusion in RGB Images

**DOI:** 10.3390/s25010088

**Published:** 2024-12-27

**Authors:** Huimin Pan, Yuting Cai, Jiayi Yang, Shaojia Niu, Quanli Gao, Xihan Wang

**Affiliations:** School of Computer Science, Xi’an Polytechnic University, Xi’an 710600, China; 230711005@stu.xpu.edu.cn (H.P.); 230711004@stu.xpu.edu.cn (Y.C.); 230721097@stu.xpu.edu.cn (J.Y.); 230721095@stu.xpu.edu.cn (S.N.)

**Keywords:** interacting hand reconstruction, feature fusion, MANO

## Abstract

Interacting hand reconstruction presents significant opportunities in various applications. However, it currently faces challenges such as the difficulty in distinguishing the features of both hands, misalignment of hand meshes with input images, and modeling the complex spatial relationships between interacting hands. In this paper, we propose a multilevel feature fusion interactive network for hand reconstruction (HandFI). Within this network, the hand feature separation module utilizes attentional mechanisms and positional coding to distinguish between left-hand and right-hand features while maintaining the spatial relationship of the features. The hand fusion and attention module promotes the alignment of hand vertices with the image by integrating multi-scale hand features while introducing cross-attention to help determine the complex spatial relationships between interacting hands, thereby enhancing the accuracy of two-hand reconstruction. We evaluated our method with existing approaches using the InterHand 2.6M, RGB2Hands, and EgoHands datasets. Extensive experimental results demonstrated that our method outperformed other representative methods, with performance metrics of 9.38 mm for the MPJPE and 9.61 mm for the MPVPE. Additionally, the results obtained in real-world scenes further validated the generalization capability of our method.

## 1. Introduction

Three-dimensional hand reconstruction is essential for various applications, including VR/AR, autonomous driving, digital medicine, and holography. In recent years, single-hand reconstruction has achieved significant success [1,2,3,4,5,6,7,8,9,10,11,12,13,14]. Many researchers have directly applied the single-hand reconstruction method to the work of both hands [15,16,17,18,19]. However, during the research process, it was found that interacting hand reconstruction presents greater challenges. First, the similarity of the appearance of both hands confuses the feature extractor, making it difficult to distinguish the features of both hands. Second, severe occlusion between two hands leads to misalignment between the hand grid and the input image. Meanwhile, it is difficult for the network to model the complex spatial relationships between interacting hands. Moon et al. [18] proposed the first large-scale two-handed interaction RGB dataset, InterHand2.6M, and many researchers have begun to experiment with reconstructing interacting hands from monocular RGB images. Inspired by existing single-hand frameworks [2,3], researchers utilized heatmaps to estimate hand joint positions [15,16,17,18,19] and re-optimize projection errors [19], which mitigated occlusion and similarity interference. However, the complex spatial relationships between interacting hands remain inadequately represented. Hampali et al. [20] applied a Transformer architecture to model interactions between the hands, but the estimated hand poses aligned poorly with the images. Later research [21,22] successfully regressed the vertices of both hands using graph convolutions, but the reconstructed hand meshes exhibited unnatural artifacts in the cases of severe occlusion. In summary, the challenges outlined above remain largely unresolved.

In this paper, we propose a novel multilevel feature fusion interaction network (HandFI) for interacting hand reconstruction based on RGB images. We designed two modules within the network. The first module is the hand feature separation (HFS) module, which utilizes attention and position encoding to efficiently focus on the key parts of the left-hand and right-hand features and maintain the spatial relationships of the features, thus distinguishing between left-hand and right-hand features. The second module is the hand fusion and attention (HFA) module, which includes the hand feature fusion (HFF) module for fusing multi-scale hand features and the interacting hand cross-attention (ICA) module for modeling spatial relationships between interacting hands. The HFF module progressively integrates multi-scale hand features, allowing the dense grid to focus on local image features, resulting in improved vertex–image alignment. The ICA module employs interacting hand cross-attention, allowing each hand to implicitly obtain the vertex features of the other hand, thereby modeling the spatial relationships between interacting hands. Extensive experiments demonstrated that our method performed exceptionally well on the InterHand 2.6M, RGB2Hands, and EgoHands datasets, yielding high-quality hand reconstruction results in real-world scenarios. In summary, our contributions are as follows:

(1) We propose a new network, HandFI, designed to reconstruct the interacting hands from RGB images and handle the interaction relationship between hands under occlusion well.
(2) We designed two modules within the network: the hand feature separation (HFS) module and the hand fusion and attention (HFA) module. The HFS utilizes attention mechanisms and positional encoding to distinguish the features of both hands while preserving their spatial relationships. The HFA aligns hand meshes with images by fusing multi-scale hand features (via HFF) and incorporates interacting hand attention (via ICA) to model the spatial relationships between the interacting hands, thereby enhancing the accuracy of hand reconstruction.
(3) Extensive experiments and qualitative analyses demonstrated that our proposed HandFI network significantly outperformed other approaches on the InterHand 2.6M, RGB2Hands, and EgoHands datasets, while also exhibiting strong generalization ability in real-world scenes.

## 2. Related Works

### 2.1. Single-Hand Reconstruction

Since the last century, there has been significant interest in hand reconstruction and gesture recognition. With the continuous development of deep learning, single-hand reconstruction [1,2,3,4,5,6,7,8,9,10,11,12,13,14] has achieved tremendous success. Zhang et al. [14] utilized shape and relative 3D joint angles to parameterize a 3D hand model for hand mesh recovery. Boukhayma et al. [1] introduced the first end-to-end deep learning approach using 2D and 3D keypoints as weak supervision for hand pose reconstruction. Baek et al. [8] incorporated 2D segmentation masks and skeletons as additional weak labels during training using a neural renderer. Chen et al. [2] proposed the first self-supervised 3D hand reconstruction method, which generates 3D meshes using semantic relationships between joints and 2D cues and completes a camera space 3D mesh recovery by aligning the generated 3D meshes with the 2D cues. Zhou et al. [3] developed a learning framework that simultaneously utilizes both 2D and 3D data to achieve efficient hand shape and pose estimation, and enhances the prediction of 3D joint positions and rotations through an inverse kinematics module. Additionally, Tang et al. [4] proposed a three-stage pipeline approach comprising joint prediction, mesh generation, and refinement to achieve high-quality alignment between hand meshes and images, while also supporting real-time prediction. Recently, Jiao et al. [5] introduced state space models into hand mesh reconstruction for the first time, presenting a lightweight and efficient 3D hand mesh reconstruction network. Although these methods have made significant progress in single-hand reconstruction, they are specifically designed for single-hand tasks and are less effective for reconstructing both hands.

### 2.2. Interacting Hand Reconstruction

Interacting hand reconstruction has proven to be a particularly challenging problem. Previous studies predominantly relied on depth cameras [23,24,25,26,27] and multi-view cameras [28,29,30], which are both expensive and time-consuming. With the introduction of the RGB dataset InterHand2.6M [18] for hand interaction, researchers [15,16,17,19,20,21,22,27,31,32] began exploring the reconstruction of interacting hands from RGB images. Some researchers [15,16,17,18] used heatmaps to estimate hand joint positions. For instance, Zhang et al. [15] employed heatmaps as attention maps to extract features from hand images and estimate hand poses. Wang et al. [27] developed a novel multi-task CNN that can capture hand movements in real-time with a single RGB camera and regress multiple information types, including segmentation, 3D hand model matching, and relative depth. Fan et al. [16] reconstructed 3D hand poses using pixel-level semantic segmentation masks and visual feature volumes. Rong et al. [19] re-optimized projection errors, but still struggled with effectively capturing dense interaction contexts. Jiang et al. [32] used Transformers to design anchors with adaptive local features and positioned them in 3D space. Hampali et al. [20] applied Transformer architecture to model interactions between two hands, but issues with aligning estimated hand poses with images remains unresolved. Recently, some researchers [21,22] successfully used graph convolution to reconstruct interacting hands, though the approach is less robust under severe occlusions, and the reconstructed meshes can suffer from unnatural deformations.

## 3. Method

### 3.1. System Override

**Figure 1:**
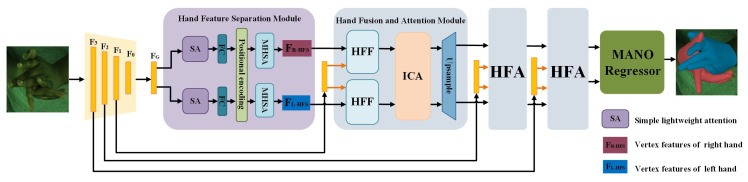
HandFI network framework: Given an RGB image of two interacting hands as the input, our network first extracts multi-scale global feature vectors. Then, it directly regresses the 3D coordinates of the surface vertices of both hands through the hand feature separation (HFS) module and three hand fusion and attention (HFA) modules. The HFA module consists mainly of the hand feature fusion (HFF) module and the interacting hand cross-attention (ICA) module.

In this paper, we propose a multilevel feature fusion interaction network (HandFI) for interacting hand reconstruction based on RGB images. As shown in Figure 1, we employed an encoder–decoder network, where the encoder extracts multi-scale features of the hand from a single RGB image. Two modules were designed based on this architecture: the two-hand feature separation (HFS) module and the hand fusion and attention (HFA) module. The highest-level feature maps obtained from the encoder stage are fed into the HFS module, which employs lightweight attention, positional encoding, and multi-head self-attention (MHSA) to make the left-hand and right-hand features, respectively, better separated from the global features while preserving the spatial relationships of the features. The HFA consists of two sub-modules: the hand feature fusion (HFF) module, which emphasizes local vertex features, and the interacting hand cross-attention (ICA) module, which learns the interaction features of the hand. The HFA is executed three times, progressively refining the hand mesh from coarse to fine. To construct the mesh topology from coarse to fine, we constructed $N_{m}$ = 3 layers of sub-mesh with $N_{1}$ = 63, $N_{2}$ = 126, and $N_{3}$ = 252 vertices, preserving the topological relationships between neighboring layers for upsampling. After the execution of the final HFA block, a linear layer was used to upsample the final sub-mesh ($N_{3}$ = 252) to the complete MANO mesh (*N* = 778), which resulted in the final vertices for both hands. Briefly, our network takes RGB images of interacting hands as the input and first extracts multi-scale global feature vectors using ResNet50. Subsequently, the 3D coordinates of the vertices on the surface of both hands are asymptotically regressed by the hand feature separation (HFS) module and the three hand fusion and attention (HFA) modules, and the interacting hands are reconstructed by the MANO regressor. The hand feature separation (HFS) module and the hand fusion and attention (HFA) module are discussed in detail in Section 3.2 and Section 3.3.

### 3.2. Hand Feature Separation Module

We adopted ResNet-50 as the encoder. The RGB image of a single interacting hand is sent to the encoder to obtain the multi-scale features of the hand ${{\{F}_{i}\in R^{C_{i}\times H_{i}\times W_{i}}\}}_{i=0}^{i=3}$, where N denotes the number of feature scales of the hand, and $C_{i}$, $H_{i}$, and $W_{i}$ denote the channel dimensionality, height, and width of the $i^{th}$ feature map, respectively. To avoid confusion between two-hand features, the left hand and right hand should have their unique features, so we introduced a simple lightweight attention (SA) in the HFS to separate the features of both hands from the global feature map, and we took the highest-level hand feature $F_{0}$ as the global feature $F_{G}$ and fed it into the SA attention to obtain $F_{left}$ and $F_{right}$:

(1) $$F_{left}=SA\left(F_{G}\right)\;F_{right}=SA(F_{G})$$

Then, we used fully connected (FC) layers to map the separated left-hand and right-hand features into feature vectors that can be shared across vertices. We concatenated the dense matching encoding $c_{i}$ of the $i^{th}$ vertex with the shared vectors to form the vertex features for both the left hand and right hand. Subsequently, the vertex features are processed through the MHSA to obtain attention-enhanced left-hand and right-hand vertex features $F_{L-HFS}^{i}$ and $F_{R-HFS}^{i}$. The MHSA effectively captures the contextual relationships between the left-hand and right-hand features by adaptively weighting the input features. Take the right hand as an example:

(2) $$F_{VR}^{i}=concat\left({FC(F}_{right}),c_{i}\right)$$

(3) $$F_{R-HFS}^{i}=MHSA(F_{VR}^{i})$$

$$i=1,\;2,\ldots ,N_{0}$$

where $N_{0}$ = 63 is the number of coarse sub-mesh vertices.

### 3.3. Hand Fusion and Attention Module

#### 3.3.1. Hand Feature Fusion Module

**Figure 2:**
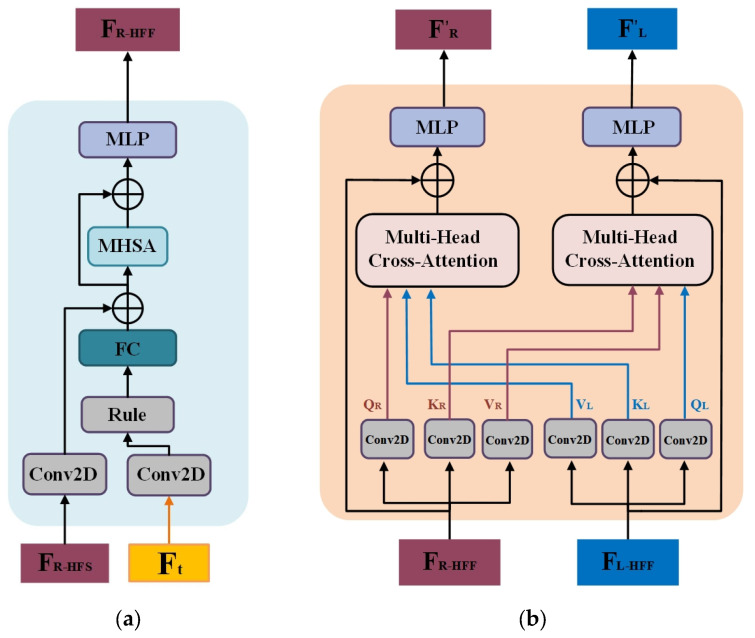
Two submodules of the HFA module: (**a**) hand feature fusion module; (**b**) interacting hand cross-attention module.

Achieving pixel alignment with the input image is challenging when the grid model is reconstructed solely using a single global feature [33]. Consequently, the network must emphasize local vertex features. Low-resolution image features mainly have global and overall structural information, while high-resolution image features focus more on local details and texture information. Therefore, incorporating multi-scale features enables sparse meshes to focus on global image features, while dense meshes focus on local image features, resulting in better vertex image alignment. To realize this preconception, we progressively inserted hierarchical image features to ensure better mesh image alignment in both local and global environments. Note that each image feature is a multi-scale feature obtained from the encoder stage. The image feature map $F_{t}$ is uniformly divided into $N_{t}\times N_{t}$ image patches on the $t^{th}$ block of the HFA block. These image patches are then compressed and flattened by a linear layer to obtain a series of feature vectors $F_{I}^{t}$. We obtained $F_{L-HFS}^{t}$ and $F_{R-HFS}^{t}$ by superimposing $F_{L-HFS}^{i}$ and $F_{R-HFS}^{i}$. Subsequently, the image features $F_{I}^{t}$ are fused with the left-hand and right-hand vertex features $F_{L-HFS}^{t}$ and $F_{R-HFS}^{t}$ and fed into the MHSA module (see Figure 2a). This process produces the attention-enhanced left-hand and right-hand vertex features $F_{L-HFF}^{t}$ and $F_{R-HFF}^{t}$, allowing the network to focus more on the edge local vertex information within the image features. Formally,

(4) $$F_{L-HFF}^{t}=MHSA(concat(F_{L-HFS}^{t},F_{I}^{t}))\;F_{R-HFF}^{t}=MHSA(concat(F_{L-HFS}^{t},F_{I}^{t}))$$

#### 3.3.2. Interacting Hand Cross-Attention Module

Previous work [15] demonstrated that the two interacting hands are correlated; therefore, the interacting hand context is important for two-hand reconstruction. We designed the interacting hand cross-attention module to implicitly represent the correlations of interacting hands. For simplicity, we denote $F_{L-HFF}^{t}$ and $F_{R-HFF}^{t}$ by $F_{L}$ and $F_{R}$, respectively. As shown in Figure 2b, we extracted the query ($Q_{h}$), key ($K_{h}$), and value features ($V_{h}$) (h ∈ L, R) for each hand using two 1 × 1 convolution layers. We then used a multi-head cross-attention mechanism to facilitate message passing between the hands. Specifically, the query features $Q_{h}$ from one hand are utilized in the multi-head attention (MHA) to obtain the key features $K_{h}$ and value features $V_{h}$ of the other hand. The bidirectional interactions of Q, K, and V between $F_{L}$ and $F_{R}$ enable the module to uncover strong correlations between the interacting hands, effectively modeling the spatial relationship between hands. The computational process is as follows:

(5) $$F_{L_{R}}=softmax(\frac{Q_{L}K_{R}^{T}}{\sqrt{d}})V_{R}\;F_{R_{L}}=softmax(\frac{Q_{R}K_{L}^{T}}{\sqrt{d}})V_{L}$$

where $F_{L_{R}}$ and $F_{R_{L}}$ denote the interacting hand cross-attention features after completing the message passing, and d is the normalization constant. Then, the interacting hand cross-attention features are fused with the hand vertex features through an MLP layer to enhance the feature representation:

(6) $$F_{L}^{\prime }=MLP\left(F_{L}+F_{L_{R}}\right)\;F_{R}^{\prime }=MLP(F_{R}+F_{R_{L}})$$

where $F_{L}^{\prime }$ and $F_{R}^{\prime }$ are the hand vertex features output from the current HFA block, which are combined with the next hierarchical image feature $F_{t+1}$ and fed into the next HFA block.

### 3.4. Loss Functions

Our loss function consists of three parts: (1) vertex loss, (2) joint loss, and (3) mesh smooth loss.

**Vertex loss:** We employed the widely used MAE loss to supervise the 3D coordinates of the hand vertices and used the MSE loss to supervise the 2D projections of the vertices. The formula is as follows:

(7) $$L_{V}=\sum_{h=L,R}\sum_{i=1}^{N}||V_{h,i}^{3D}-V_{h,i}^{3D,gt}{||}_{1}+||V_{h,i}^{2D}-V_{h,i}^{2D,gt}{||}_{2}^{2}$$

where $V_{h,i}$ denotes the $i^{th}$ vertex.

**Joint loss:** The predicted hand vertices are multiplied by a predefined joint regression matrix J to derive the positions of the hand joints. This is computed by using the following equation:

(8) $$L_{J}=\sum_{h=L,R}\sum_{i=1}^{N}||{JV}_{h,i}^{3D}-JV_{h,i}^{3D,gt}{||}_{1}+\sum_{h=L,R}\sum_{i=1}^{V}||{JV}_{h,i}^{2D}-{JV}_{h,i}^{2D,gt}{||}_{2}^{2}$$

**Mesh smooth loss:** We used two different smoothing losses to ensure the geometric smoothness of the predicted vertices. We used the face normal loss:

(9) $$L_{n}=\sum_{h=L,R}\sum_{f=1}^{F}\sum_{e=1}^{3}\|e_{f,i,h}\cdot n_{f,h}^{gt}{\left|\right|}_{1}$$

where $n_{f}^{gt}$ is the normal vector of the face f computed from the ground truth mesh. In addition, we introduced the edge length consistency loss:

(10) $$L_{e}=\sum_{h=L,R}\sum_{i=1}^{E}\|e_{i,h}-e_{i,h}^{gt}{\left|\right|}_{1}$$

## 4. Experiments

### 4.1. Datasets, Metrics, and Implementation Details

**InterHand2.6M dataset:** InterHand2.6M [18] is a large-scale dataset containing 2.6 million hand images, covering a variety of single-hand and interacting hand poses with realistic mesh annotations. We used the 5FPS version of the dataset published in [18]. Since our approach focused only on the reconstruction of interacting hands, we selected the interacting hand data therein, specifically including 366 K training samples and 261 K test samples.

**RGB2Hands and EgoHands datasets:** The RGB2Hands [27] dataset consists of four different types of two-handed interaction video sequences and 1724 RGB frames, while the EgoHands [34] dataset consists of egocentric two-handed interaction video sequences and 4800 RGB frames from 48 different scenes. Since our method targets RGB images, we used RGB frames from both datasets as datasets. We conducted qualitative experiments on both datasets. We conducted qualitative experiments on these two datasets. The variety of scenarios in these datasets provided a comprehensive evaluation of our approach.

**Metrics:** To assess the accuracy of the reconstructed hand posture and shape, we used two types of positional errors in millimeters: mean per joint position error (MPJPE) and mean per vertex position error (MPVPE). Additionally, we used the percent correct keypoint (PCK) curves in the range of 0 to 50 mm for the evaluation.

**Implementation details:** We implemented our network using PyTorch (version: 1.8.1) and trained and evaluated it on an NVIDIA RTX 3090 GPU (NVIDIA, Santa Clara, CA, USA). During the training process, we chose the Adam optimizer [25], set the initial learning rate to 1 × 10^−4^, and employed a cosine annealing learning rate strategy. The entire training period consisted of 60 epochs. To improve the model’s generalization ability, we applied a series of data augmentation techniques, including random rotation, translation, scaling, and motion blur. During the data preprocessing, we performed the 2D projection of hand vertices, cropped the hand regions, and resized them to a resolution of 256 × 256.

### 4.2. Qualitative Results

**Figure 3:**
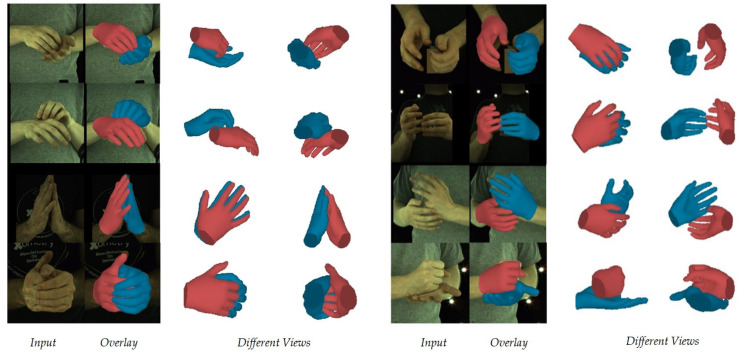
Qualitative results of our method on the InterHand2.6M dataset.

**Figure 4:**
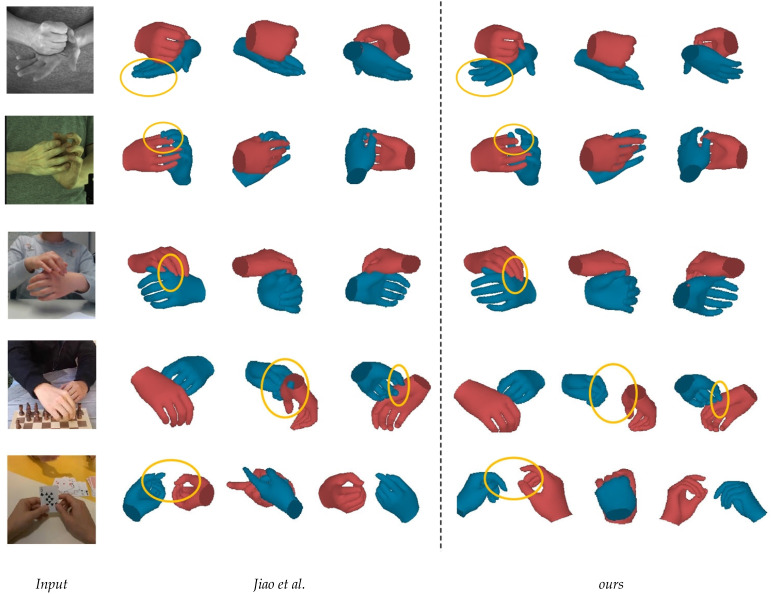
Qualitative comparison of our method with Jiao et al. [22] on the InterHand2.6M dataset (rows 1–2), RGB2Hands dataset (row 3), and EgoHands dataset (rows 4–5). Our method generated more accurate results, whereas Jiao et al.’s method resulted in more collisions (row 2) and errors in calculating the relative depth between the hands (row 4). In cases of severe occlusion, their method also generated incorrect hand gesture estimations (row 5).

The qualitative results of our method on the InterHand2.6M dataset are presented in Figure 3. Our method demonstrated excellent reconstruction capabilities, even in the presence of severe occlusions and diverse hand interactions. Additionally, we performed a qualitative comparison with method [22] across the InterHand2.6M, RGB2Hands, and EgoHands datasets. Notably, our method achieved more accurate reconstruction results, fewer collisions between the hands, and an accurate calculation of the relative depth between the hands (see Figure 4).

**Figure 5:**
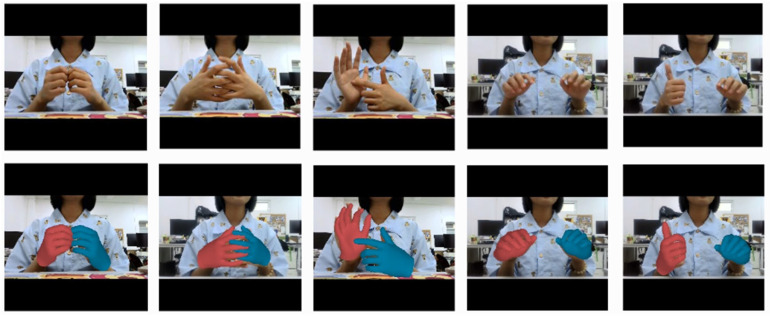
Results of our method in real-world scenes.

Additionally, our method can be extended to real-world scenes that were not included in the training. Figure 5 illustrates that our method delivered outstanding results with typical real data, indicating robust generalizations for handling finger interactions, hand collisions, and various hand poses. During the inference, our model could run at 30–35 fps on a single NVIDIA RTX 3090 GPU. This result opens up possibilities for future applications in real-time interactive scenarios.

### 4.3. Quantitative Comparisons

**Figure 6:**
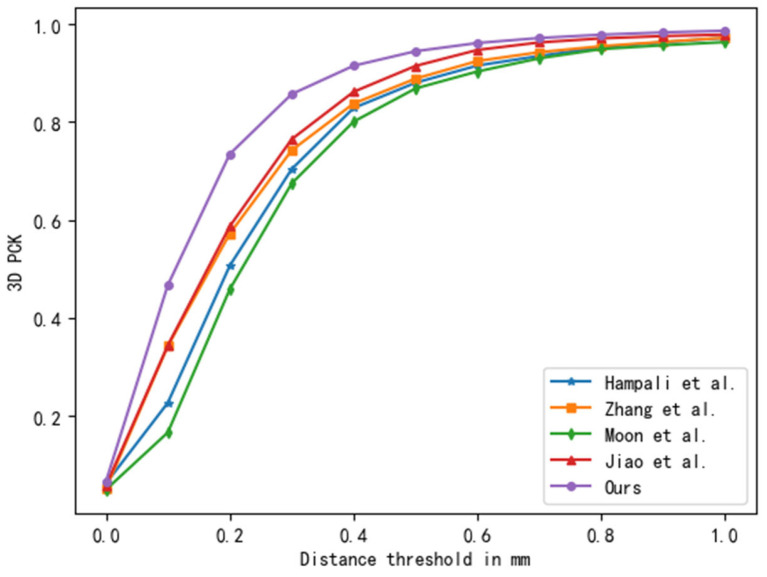
Comparison with other methods on the InterHand2.6M dataset. ‘Hampali’ refers to reference [20], ‘Zhang et al.’ refers to reference [15], ‘Moon’ refers to reference [18], ‘Jiao’ refers to reference [22].

We compared our method with previous state-of-the-art single-hand reconstruction and two-hand pose estimation methods, as shown in Table 1. The first two rows of the table present the reconstruction performance of single-hand methods [1,3], which indicates that the direct application of single-hand reconstruction methods did not yield satisfactory results due to severe self-occlusion and appearance similarity. We then compared our method with recent two-hand reconstruction methods [15,16,18,20,22,32,35,36,37,38], as shown in rows 3–8 of Table 1. Our approach significantly reduced the MEJPE and MEVPE, which was attributed to the successful separation of hand features, the effective fusion of multi-scale features, and the effective modeling of the spatial relationships of interacting hands. Compared with a representative model [15], our model size was reduced by 76.4% and both the MJPE and MVPE were reduced by 30%. Figure 6 depicts the PCK curves of our method at 0–50 mm error thresholds, further demonstrating the excellent performance across all error threshold levels.

### 4.4. Ablation Study

**Baseline:** We progressively regressed the parameters of the MANO hand model by directly using the global features extracted from the dual-hand encoder, and then input these parameters into a regressor to obtain the output for both hands. The results show that directly regressing the hand parameters yielded suboptimal performance.

**Figure 7:**
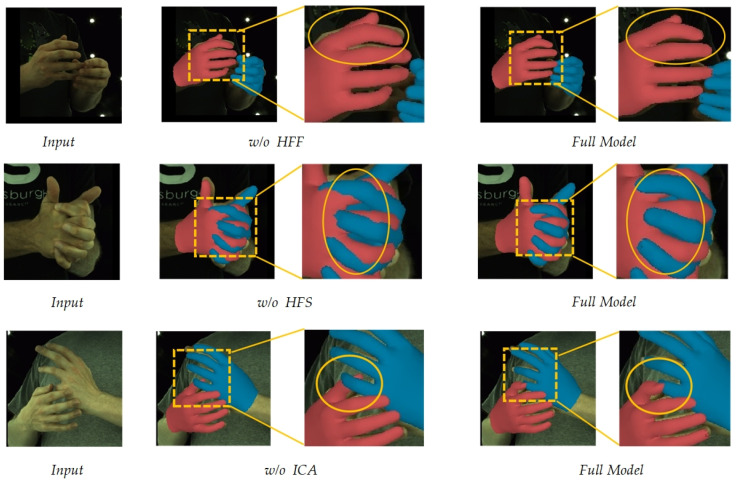
Qualitative ablation results on InterHand2.6M. ‘w/o’ represents the removal of a specific module from the model.

**Adding modules:** On the basis of the baseline, we first integrated the HFF module to better focus on the vertex features, which allowed the dense mesh to attend to local image features. As shown in Table 2, adding the HFF improved the MEJPE and MEVPE by about 1.4 mm, demonstrating that the HFF could produce better vertex–mesh alignment. Next, we incorporated the HFS module, which further enhanced the MEJPE and MEVPE by 0.17 mm, validating the HFS’s ability to differentiate hand features. Finally, we added the ICA module to model the hand context, which achieved an approximately 0.5 mm improvement in both metrics, confirming that the ICA could exploit the correlations between the two hands and address the occlusion challenges. Figure 7 presents the qualitative results of each module, providing a more intuitive perspective on their respective effects.

## 5. Conclusions

In this paper, we propose an interacting hand reconstruction network based on RGB images called HandFI. By introducing the HFS module, we effectively utilized the attentional mechanism and positional coding to distinguish between left- and right-hand features while maintaining the spatial relationship between them. In addition, the HFA module integrated multi-scale hand features through the HFF sub-module, which greatly improved the alignment accuracy of the hand vertices with the input image. The ICA sub-module could effectively simulate the complex spatial relationships between interactive hands. The experimental results show that our method performed excellently on the InterHand2.6M, RGB2Hands, and EgoHands datasets, as well as in untrained real scenarios. Ablation studies confirmed the effectiveness of the HFS, HFF, and ICA modules. However, our model does not explicitly address mesh collisions, so occasional penetration occurred in the cases of severe hand crossing. Future work will concentrate on employing a perspective camera model for depth inference and translation simulation to mitigate this issue.

## Figures and Tables

**Table 1:** Comparison of the MPJPE (mm) and MPVPE (mm) on the InterHand2.6M dataset. The single-hand reconstruction results were from [15], and some of the two-hand reconstruction results were from [20].

	MPJPE↓	MPVPE↓
Zhou et al. [3] (2020)	23.48	23.89
Boukhayma et al. [1] (2020)	16.93	17.98
Moon et al. [18] (2020)	16.02	-
Fan et al. [16] (2021)	14.27	-
Zhang et al. [15] (2021)	13.48	13.95
Kim et al. [17] (2021)	12.08	-
Meng et al. [38] (2022)	13.01	-
Hampali et al. [20] (2022)	14.34	14.36
Di et al. [35] (2022)	12.56	12.37
Jiang et al. [32] (2023)	10.96	-
Moon et al. [36] (2023)	11.12	13.01
Jiao et al. [22] (2023)	13.15	13.52
Zhang et al. [37] (2024)	13.25	13.91
**Ours**	**9.38**	**9.61**

**Table 2:** The result of ablation study on InterHand2.6M.

	MPJPE↓	MPVPE↓
Baseline	11.45	11.73
Baseline + HFF	10.05	10.30
Baseline + HFF + HFS	9.88	10.13
Baseline + HFF + HFS + ICA (ours)	**9.38**	**9.61**

## Data Availability

Here, we provide links to the three datasets used in our experiments. InterHand 2.6M: https://mks0601.github.io/InterHand2.6M/ (accessed on 26 April 2024); RGB2Hands: https://dl.acm.org/doi/abs/10.1145/3414685.3417852 (accessed on 24 July 2024); EgoHands: https://vision.soic.indiana.edu/projects/egohands/ (accessed on 15 August 2024).
